# Causal machine learning methods for understanding land use and land cover change

**DOI:** 10.1007/s10980-025-02279-7

**Published:** 2025-12-28

**Authors:** F. Eigenbrod, Peter Alexander, Nicolas Apfel, Ioannis N. Athanasiadis, Thomas Berger, James M. Bullock, Gregory Duveiller, Julian Equihua, Isaura Menezes, Rodrigo Moreira, Dilli Paudel, Vasileios Sitokonstantinou, Markus Reichstein, Simon Willcock, Tamsin Woodman

**Affiliations:** 1 School of Geography and Environmental Science, University of Southampton, Southampton, SSO17 1BJ UK; 2 School of Geosciences, University of Edinburgh, Edinburgh, EH9 3JW UK; 3 Department of Economics, University of Innsbruck, 6020 Innsbruck, Austria; 4 Artificial Intelligence, Wageningen University and Research, Wageningen, The Netherlands; 5 Department of Land Use Economics, University of Hohenheim, 70599 Stuttgart, Germany; 6 UK Centre for Ecology & Hydrology, Wallingford, OX10 8BB UK; 7 Max Planck Institute for Biogeochemistry, 07745 Jena, Germany; 8 Department of Computational Landscape Ecology, Helmholtz Centre for Environmental Research (UFZ), 04318 Leipzig, Germany; 9 Environmental Sciences Graduate Program, Federal University of Rondônia, Ji-Paraná, RO 76900-726 Brazil; 10 Image Processing Laboratory, Universitat de València, Paterna, 46980 València, Spain; 11 School of Environmental and Natural Sciences, Bangor University, Bangor, Gwynedd, LL57 2DG UK; 12 School of Biological Sciences, University of Aberdeen, Aberdeen, AB24 2TZ UK

**Keywords:** Land use change, Deforestation, Agricultural expansion, Machine learning, Socio-ecological systems, Complex systems

## Abstract

**Context:**

Understanding the roles of different drivers in land use and land cover change (LULCC) is a critical research challenge. However, as LULCC is the result of complex, socio-ecological processes and is highly context dependent, achieving such understanding is difficult. This is particularly true for causal modelling approaches that are critical for effective policy formulation. Causal machine learning (ML) methods could help address this challenge, but are as yet poorly understood or applied by the LULCC community.

**Objectives:**

To provide an accessible introduction to the state of the art for causal ML methods, their limitations, and their potential applications understanding LULCC.

**Methods:**

We conducted two workshops where we identified the most promising ML methods for increasing understanding of LULCC dynamics.

**Results:**

We provide a brief overview of the challenges to causal modelling of LULCC, including a simple example, and the most relevant causal ML approaches for addressing these challenges, as well as their limitations.

**Conclusions:**

Causal ML methods hold considerable promise for improving causal modelling of LULCC. However, the complexity of LULCC dynamics mean that such methods must be combined with domain understanding and qualitative insights for effective policy design.

## Introduction

Human transformation of terrestrial land (land use and land cover change; LULCC) has fundamentally impacted ecosystems globally (Ellis et al. 2013). Over 75% of the global terrestrial area has been transformed during the last millennium, and up to 33% of this change happened in the last 60 years (Winkler et al. 2021). Historically, these changes were largely driven by conversion of natural land cover to agriculture to provide food for a growing human population with changing diets, as well as the conversion of agricultural and natural land to urban areas. Agricultural expansion continues today (Potapov et al. 2021) but in many regions, agricultural land abandonment has also taken place, leading to major increases in forested lands (Song et al. 2018). Loss of natural land cover has, in general, had major negative implications for biodiversity (Maxwell et al. 2016) and has been a considerable source of carbon emissions (Arneth et al. 2017). As such, policies that enable and accelerate restoration of natural land cover (e.g. reforestation) are critical in halting and reversing biodiversity declines and can also have climate change co-benefits (Mori et al. 2021; Tölgyesi et al. 2025). Understanding drivers of LULCC is therefore a critical research challenge, as such understanding is required to develop such policies; it is also critical to develop credible projections of future trajectories of LULCC under different climatic and socio-economic scenarios (e.g. Brown et al. 2022).

Understanding LULCC and projecting land use outcomes of future socio-environmental changes is difficult because land use patterns are emergent properties of complex, socio-ecological systems (Berkes et al. 1998). Governance, cultural practices and technologies all shape land use, and interact with local environmental conditions and constraints in complex ways across multiple scales (Meyfroidt et al. 2022). This means predictors of LULCC are context-dependent, varying by location and scale. Such complexity is common for socio-ecological questions, with similar problems making synthesis and generalisations difficult in ecology (Spake et al. 2022). The situation is further complicated by the changing nature of socio-ecological interactions – a situation that is likely to accelerate as the impacts of climate change and biodiversity loss increasingly affect both biophysical and socio-economic systems globally (Pedde et al. 2021). The relative importance of different predictors of LULCC are therefore likely to evolve over time (e.g. Eigenbrod et al. 2020), Moreover, historic land use changes may have led to irreversible transitions, limiting options for future changes (Meyfroidt et al. 2022). This complexity matters not only because it makes understanding LULCC difficult per se, but also as it makes designing policies to achieve socially and ecologically beneficial LULCC challenging (Meyfroidt et al. 2022). By contrast, detecting changes in land cover has seen an immense progress in the past years driven by progress in remote sensing, computer vision and data science, leading to a proliferation of global, high resolution, multi-year spatial LULCC datasets derived from remote sensing (Bürgi et al. 2022; Reynolds et al. 2025).

To date, researchers have addressed the complexity of understanding LULCC with a combination of detailed case studies and process-based and statistical modelling approaches (Verburg et al. 2019; Cabral et al. 2024). This has led to the development of general theory (e.g. forest transition theory; Mather 1992) and numerous newer theories on LULCC (reviewed in Meyfroidt et al. 2018, 2024). It has also led to list of key variables driving of different types of LULCC (see Seymour & Harris (2019)for a review on deforestation and Plieninger et al., (2016) for a review of drivers of LULCC in Europe). However, specific understanding of which theory, or which sets of variables are most important in a particular setting remains lacking in most locations, hampering effective policy design. Data-driven methods like machine learning (ML) and deep learning (DL) are also increasingly used to create LULCC datasets from remote sensing (reviewed in Vali et al. 2020) and to predict LULCC at local and regional scales. However, ML/DL approaches primarily rely on statistical correlations, and are therefore poorly suited to predicting LULCC under changing socio-ecological conditions (e.g. under climate change) (reviewed in Wang et al., (2022)).

## Causal modelling and LULCC

**Causal modelling**, particularly **causal effect estimation** is of particular interest for effective policy formulation as it enables an understanding of how key variables (e.g. a new policy) is likely to affect future LULCC (Meyfroidt 2016; Magliocca et al. 2023). In order to understand a causal effect, one must ask a counterfactual question: what would have happened to a given outcome variable if a certain change had not taken place? Hence, a causal effect arises from the comparison of two states of the world, one in which the treatment has taken place and one in which it has not (i.e. the ‘control’).

A key aspect of any causal analysis is defining which variables need to be accounted for to achieve causal understanding. Estimating causal effects relies on making the treatment and control groups comparable; typically by adjusting for observable confounding variables – factors that affect both the outcome and the likelihood of receiving the treatment. Statistical matching approaches are commonly used to ensure that control and treatment units are similar in every respect except the treatment variable, thereby constructing a meaningful comparison (counterfactual)(Magliocca et al. 2023). For example, examination of the effectiveness of protected areas requires ensuring that control and treatment sites are similar in terms of key confounders such as distance to population centres (Schleicher et al. 2020), as more isolated locations are usually less likely to undergo LULCC (e.g. deforestation). In social sciences (including economics), such matching is often done using panel data (where multiple datasets are available over time), and causal modelling often follows the **potential outcomes framework** (Rubin 2005). Applications of this counterfactual approach to causal modelling – also called the Rubin Causal Model approach – are reviewed by Magliocca et al. (2023) in the context of LULCC.

Causal approaches have led to important specific insights in understanding LULCC (e.g. in the effectiveness of protected areas in stopping deforestation (Andam et al. 2008) or the role of conflict in deforestation (Christiansen et al. 2022)), but robust implementations remain relatively rare (reviewed by (Meyfroidt 2016; Bürgi et al. 2022; Magliocca et al. 2023). A key reason is likely the lack of high-quality counterfactual data on policy “experiments”—for example panel data covering the period before and after new legislation on deforestation is put in place—and where such data do exist, they tend to be highly localized and often lack external validity, limiting their utility in increasing general understanding.

**Fig. 1:**
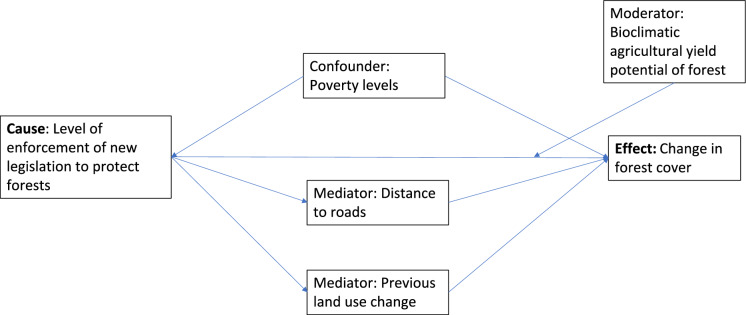
A simplified Directed Acyclic Graph (DAG) for an example of a causal model for LULCC (deforestation). *Confounder*: A variable that influences both the cause and outcome effect. *Mediator*: a variable on the causal path affected by the cause that affects the effect, when some or all of the causal effect is indirect. *Moderator*: A variable that influences the direction and/or strength of the relationship between the treatment (cause) and the outcome (effect) (Van Cleemput et al. 2025)

Another, important reason for the relative lack of progress in causal modelling for LULCC is likely the sheer complexity of the human-natural linkages that determine LULCC outlined earlier, which make isolating causal factors extremely challenging (Ferraro et al. 2019). Indeed, in **complex systems** such as those driving LULCC, chains of linked causal effects (causal chains) are often necessary to meaningfully understand underlying causal mechanisms. Causal diagrams, or **directed acyclic graphs (DAGs**), offer an intuitive approach to organize our thinking about these causal structures and how variables are interconnected (Pearl 2000). DAGs serve as helpful visual tools for clarifying assumptions, identifying potential sources of bias and guiding analytical strategies, and often inform  **structural causal models **(SCM). DAGs appear particularly well-suited to LULCC research, where complex socio-environmental interactions are at play. Despite overlapping goals, the DAG-based causal inference framework and the potential outcomes framework have largely developed in parallel and remain only partially integrated in practice. We outline a greatly simplified example of a DAG to illustrate the approach (Fig. 1). Recent work (Van Cleemput et al. 2025) outlining how causal inference methods can be combined with ‘big’ remote sensed derived data to increase desirable ecological outcomes in landscapes is also highly relevant to LULCC as it focuses on complex, multi-scale socio-ecological terrestrial systems, and provides an excellent accessible example of a more detailed DAG relevant to LULCC (spread of invasive trees).

Despite its potential, using DAGs to move beyond correlations and answer counterfactual questions is not straightforward, as one must make certain assumptions. These include that there are no hidden confounders, that there are no feedback cycles, that the causal relationships represented in the DAG are accurate, and the observed outcomes align with the treatment under study (consistency) (Pearl 2000). Additionally, we assume exchangeability, meaning that units with the same set of covariates are comparable. These assumptions are challenging for complex systems such as LULCC. For example, it is straightforward to come up with a simplified example of a DAG for LULCC that illustrates some of the relationships (e.g. Figure 1); the challenge lies in capturing the full complexity of all the relevant socio-ecological processes that lead to LULCC (Wang et al., (2022).

**Fig. 2:**
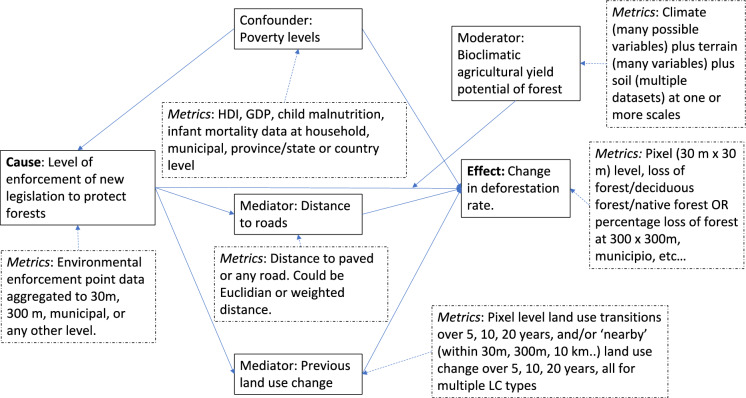
The multiple plausible metrics (dashed boxes) for the variables (solid boxes) in the simple DAG of deforestation outlined in Fig. 1

Implementation of DAGs for causal analyses becomes even more difficult as the amount of spatial data relevant to LULCC increases. This is because the ways that variables can be combined (the ‘dimensionality’ of the data) increase exponentially as the number of potential variables increases. ‘Big data’ tends to have ‘high dimensionality’, as it includes a large number of variables. This makes finding causal variables more challenging and leads to other statistical issues collectively known as ‘*the curse of dimensionality’* (Altman and Krzywinski 2018). The ‘curse’ is problematic for complex interdisciplinary problems such as LULCC as the different social, economic and ecological factors interact with each other in complex ways. The best way of addressing these problems to answer causal questions is to use domain understanding to select key variables based on clear, a priori predictions (Altman and Krzywinski 2018). However, in LULCC this is challenging as there are many variables that could be important. It is also often the case that there are multiple, plausible metrics for a given variable. We briefly outline this problem by illustrating a few of the different ways that the variables in our simple exemplar DAG (Fig. 1) can be measured (Fig. 2). A final issue that has the potential to further increase the dimensionality of LULCC causal analyses is the measurement error associated with all remote sensing based LULCC datasets. These will vary between the satellites and sensors used to create the images, and – critically – can lead to systematic bias in the response variable (LULCC) due, for example, to issues such as higher cloud cover in the tropics than in temperate systems regions. The implications of such errors due to remote sensing products for ecological causal analyses are outlined in Van Cleemput et al. (2025). In summary, given the complexity of LULCC and the additional challenges caused by the high dimensionality of LULCC analyses, it is not surprising that classical causal methods or DAGs have had only limited application to date.

## Causal machine learning and LULCC

**Causal machine learning (causal ML)** provides a set of ML methods aimed at unravelling cause-and-effect relationships within complex systems, transcending traditional correlation-based ML analyses. Causal ML is a new and rapidly evolving field, with inconsistencies in methods and language. At its core, causal ML seeks to estimate the causal effect of variables, interventions, treatments or policies on specific outcomes, while at the same time accounting for problems that arise with big data. As such, it has great promise both in enabling better understanding of the causes of LULCC dynamics, and also in using such understanding to predict how effective different policy options will be for enabling sustainable land use transitions. However, to our knowledge, causal ML methods remain poorly implemented by the LULCC modelling community, despite the considerable promise they offer.

This Perspective aims to provide an accessible introduction to the state of the art and make the LULCC community aware of causal ML techniques, their limitations, and their potential applications. It arises from two interdisciplinary three-day workshops attended by the authors in 2022 and 2023 where we set out to identify the potential of ML methods for increasing understanding of LULCC dynamics. The authors have expertise in LULCC modelling, causal ML and data science, econometrics, and landscape ecology. Box 1 provides definitions of key terms; we then use examples to describe some particularly useful potential applications of causal ML.

### Box 1 – Glossary of key terms.

**Complex systems:** Systems formed by (typically) large numbers of entities that interact dynamically, resulting in emergent properties and behaviours.

**Causal modelling:** Statistical models that enable the magnitude of cause-and effect relationships to be identified from observed association(s).

**Causal effect estimation:** A specific type of causal model that quantifies the effect of changing a particular variable (cause or treatment) on a target variable (outcome). It also allows counterfactual questions to be answered: what would have happened to a given outcome variable if a certain change (e.g. the implementation of a new policy) had not been in place?

**Potential outcomes framework:** Also known as the Rubin Causal Model, this causal modelling framework uses the counterfactuals approach to enable causal inference and is the basis of much non-experimental causal inference in social sciences. Statistical matching approaches, often based on panel data, are frequently used to control for confounding variables and enable the causal effect of a given intervention on a desired outcome variable to be identified.

**Directed acyclic graph (DAG):** A graphical representation of all presumed causal relationships between variables. Also known as causal diagrams, they serve as helpful visual tools for clarifying assumptions, identifying potential sources of bias and guiding analytical strategies for more complex systems that may involve chains of linked causal effects (causal chains), rather than causal effects of single interventions.

**Structural causal models** (SCMs)**:** A formal framework for representing causal relationships using structural equations, often informed by directed acyclic graphs (DAGs). SCMs allow the analysis of direct and indirect effects, counterfactuals and complex systems of linked causal mechanisms.

**Machine learning (ML**) methods: A family of analytical tools that enables data-driven identification of patterns within large datasets and making predictions based on historical data. ML methods excel in predicting outcomes, but (traditionally) lack causal understanding so perform poorly in novel or changing environments.

**Causal machine learning (ML)**: A subset of ML methods aimed at unravelling cause-and-effect relationships in large datasets by combining the rigour of causal modelling approaches with the flexibility and ‘big data’ handling capabilities of traditional ML methods.

**Causal discovery:** Methods for identifying plausible causal relationships between variables from data. We often use them when the structure of the causal graph (DAG) is unknown. It can be the first step in DAG-based causal inference, by enabling the subsequent estimation of causal effects once the causal structure is established.

## Causal discovery: Learning the structure of the DAG from data

**Causal discovery** refers to a set of methods aimed at learning the causal structure (i.e., the relationships between variables), directly from data, typically represented as a DAG. Unlike traditional approaches that assume a predefined causal structure (as is often the case in panel regressions or DAGs manually specified by experts), causal discovery infers the structure from observational data using algorithms. These include the PC algorithm (Spirtes and Glymour 1991) and its successors, as well as nonlinear methods like convergent cross mapping (CCM) (Runge 2023). For example, in a dataset including LULCC we might not even know whether LULCC is the origin of a causal relationship or the result, or at which scale a given variable is causal. At its simplest, causal discovery aims to identify sets of causal models that are statistically indistinguishable from each other (formally, they have the same *Markov equivalence class*) as they share common patterns of conditional independence with other variables (formally the same *d-separation patterns*) (Huber 2024). When observational data is insufficient to identify the correct causal model, the challenge of model ambiguity may be resolved by incorporating external sources of information. This can involve domain knowledge, theoretical principles, previous empirical findings, or knowledge of the temporal order of events (Huber 2024). These external insights can provide valuable context to constrain potential causal models. In practice, fully understanding all potential causal relationships for a complex DAG (e.g. for LULCC system) is challenging due to the complexities of the dependency structures of the variables (Huber 2024). Moreover, the assumptions on the functional relationships between variables and on the error terms are quite restrictive. This means causal discovery is inherently difficult, and if domain knowledge can be used to identify potential causal pathways in a DAG, this should be used rather than causal discovery. However, causal discovery methods can still be useful in such cases, as they can be used—under the appropriate assumptions—to identify all observed variables (e.g. regional GPD, topography, access, land ownership) that have an effect on an outcome variable (e.g. LULCC). This is very useful as a first step in using ML for causal effect estimation, as it already reduces the complexity and number of potential variables within a DAG. This is particularly the case in complex and dynamic systems, where the structure of the DAG may shift faster than our theoretical understanding (e.g., across space or time). Here, causal discovery can serve as a useful auxiliary tool, not to replace expert knowledge but to guide or narrow the space of plausible models. Examples (from economics) of causal discovery including R code and packages are available in the Huber (2024) review.

## Causal ML for inference: using double ML to reduce the effect of confounders in causal effect estimation

Causal effect estimation requires that the direction of causation is known – e.g. treatment D (for example regional GDP) influences outcome Y (LULCC), rather than vice versa. In order to guarantee that the analysis resembles an randomized control trial as closely as possible, the treatment and control groups need to be made comparable and to do so observable variables need to be accounted for, as discussed earlier. The key problem is that it is often unknown which variables are confounders and which function best describes their influence on D and Y; this is particularly problematic for complex issues such as LULCC. Causal discovery methods can help reduce the possible subset of variables, but, as discussed, in complex systems such as LULCC the remaining plausible subset can still be very large. Empirical researchers implicitly make assumptions on how the confounders enter the model. It is tempting to directly model the effect of a set of observable variables X on LULCC using ML methods which allow for model selection and flexible combinations of the variables. One example would be to use the Least Absolute Shrinkage and Selection Operator (LASSO) (Tibshirani 1996) to select control variables. In general, such a naïve approach will lead to unreliable (biased) results due to regularization bias, i.e. the bias arising from estimation error, and overfitting bias, i.e. the bias that arises when using the same dataset for model selection and subsequent estimation.

In response to this problem, a seminal paper by Chernozhukov et al. (2018) has proposed the use of double ML (DML), where ML estimators for the outcome and the treatment are combined in a (moment) equation that includes the treatment effect of interest and that allows for slight misspecifications of the functions that describe the true effect of the confounders on D and Y. Overfitting bias is tackled by using cross-fitting, a technique where one split of the dataset is used in the ML step and the other for estimation and the roles of the splits is reversed later on. Under assumptions which are met by many of the ML methods currently in use, such as LASSO, Ridge, Random Forest or Boosting, DML provides a decrease in bias of the causal effect estimate and therefore more reliable estimates. DML provides a general framework on how to implement ML methods when trying to estimate a causal effect and has given rise to an entire literature; see Ahrens et al. (2025) for a recent review. A recent relevant example uses a DML framework using economic panel data from 30 provinces in China over 17 years to understand the causes of urban–rural integration (Lu et al. 2025).

## Causal ML to address context dependency in LULCC: Causal forests

One case in which causal ML has particular relevance for LULCC is when the effectiveness of policy interventions varies by context, i.e. it is heterogeneous across space, time or other covariates. This is almost always the case for LULCC (Spake et al. 2019; Meyfroidt et al. 2022). For example, commodity driven deforestation is dominant in South America, while subsistence driven shifting agriculture is often, but not always, the dominant driver of deforestation in Africa (Curtis et al. 2018). In other words, we are often interested quantifying conditional treatment effects (CATE), rather than the average treatment effect (ATE) of a given policy intervention – the latter has traditionally been the main focus of causal inference. Here, causal forests (Wager and Athey 2018)—a special case of generalized random forests (Athey et al. 2019)—are applicable. These are used to understand heterogeneous treatment with big data. Causal forests build on the very widely used random forest approach and extend these to enable causal effect estimation and predict differential treatment responses by context (conditional average treatment effects). They do so by a form of orthogonalization (as is also the case for DML) where ‘baseline’ effects are removed. A relatively accessible recent overview (with examples from psychiatry) is Sverdrup et al. (2025), with the ‘grf’ R package (Tibshirani et al. 2024) also providing a good overview with worked examples. At present, most real-world examples of causal forests that are relevant to LULCC use a counterfactual, rather than a causal discovery approach. For example, causal forests were used to understand the spatial heterogeneity of crop rotation and landscape crop diversity on yields in Flanders, after accounting for factors such as climate (Giannarakis et al. 2022). A recent simulation study has shown that causal forests work well with spatial datasets (Credit & Lehnert 2024), suggesting they are well suited to LULCC questions.

## Combining causal discovery and causal inference methods

It is possible to address more complex and poorly understand causal relationships by combining causal discovery and causal ML methods focused on causal inference (DML and causal forests). For example, Soleymani et al. (2022) propose a new method that uses DML to sequentially estimate the direct effects of different candidate variables in a DAG on an outcome, controlling for all other potential variables. This latter approach is likely to be very useful for many LULCC questions, as fully understanding the DAG (via causal discovery methods) may often be necessary. A technical review of causal methods – including a detailed flow chart of the most relevant methods for different types of data – exists for time series data (Runge et al. 2023) and complements this Perspective.

## Future directions – knowledge guided machine learning

Data driven methods will never enable prediction if future LULCC is determined by different variables, or combinations of variables. This is because ML methods, like all statistical models, cannot accurately predict outside of the parameter space of the data on which they were built. Such non-analogue LULCC is extremely likely as climate change and technological changes (e.g. renewable energy) increasingly impact land use systems. A potential way of addressing this is via hybrid models that link ML techniques with process models. Reichstein et al. (2019) provide a framework for using hybrid deep learning approaches to improve models of the Earth system by linking data-driven, correlation-based models with process-based models based on causal understanding of biogeochemical cycles. This approach is more generally known as physics-guided (Willard et al. 2023) or knowledge-guided machine learning (Karpatne et al. 2017; Liu et al. 2022). Unfortunately, applying this approach LULCC is not yet possible, given that no good process models of the socio-ecological processes that characterize LULCC exist. However, such hybrid approaches may become possible in the future as our causal understanding of LULCC improves.

## Caveats and conclusions

Despite their potential, using causal ML methods to understand LULCC comes with several major caveats. Causal inference of any type will remain challenging for LULCC, due to feedbacks and complex interactions between variables (Ferraro et al. 2019). Such feedbacks mean it is very difficult to ensure that the variables affecting the treatment do not also affect the outcome (‘excludability’). For example, the potential future value of forested land for agriculture is likely to affect whether or not it is protected under legislation; it is also likely to affect whether or not legislation is effective in stopping deforestation. Such feedbacks also mean that the assumption of ‘No Interference” (e.g. the effect of an intervention on loss of forest in one region shouldn’t depend on whether the same intervention is carried out in another region”) is unlikely to hold; indeed such ‘leakage’ is common in LULCC (Meyfroidt et al. 2022). Reverse causality (where the roles of cause and effect are switched for two variables) can also be an issue for LULCC dynamics – for example increases in regional GDP could lead to deforestation, but deforestation itself could also drive increases in regional GDP. In addition, any observational study is likely to be affected by unmeasured confounding variables that cannot be controlled for, meaning conclusions need to be treated with caution (Giannarakis et al. 2022). A broader overview of key assumptions of causal ML for agriculture that will also be relevant for most LULCC is provided by Sitokonstantinou et al. (2024). While causal ML analyses of LULCC will never provide complete answers they can provide important complementary insights to more case-based site-specific approaches. In other words, causal ML is no substitute for understanding and careful study design for causal questions. This means that deeply understanding LULCC dynamics and effective policy interventions to enable sustainable LULCC pathways will require novel interdisciplinary collaborations to ensure domain understanding and qualitative insights are combined with the power of causal ML methods.

## Data Availability

No datasets were generated or analysed during the current study.
